# Preparation of Multifunctional Plasma Cured Cellulose Fibers Coated with Photo-Induced Nanocomposite toward Self-Cleaning and Antibacterial Textiles

**DOI:** 10.3390/polym13213664

**Published:** 2021-10-24

**Authors:** Hany El-Hamshary, Mehrez E. El-Naggar, Tawfik A. Khattab, Ayman El-Faham

**Affiliations:** 1 Chemistry Department, College of Science, King Saud University, Riyadh 11451, Saudi Arabia; aelfaham@ksu.edu.sa; 2 Department of Chemistry, Faculty of Science, Tanta University, Tanta 31527, Egypt; 3 Textile Research Division, National Research Center (Affiliation ID: 60014618), Cairo 12622, Egypt; ta.khattab@nrc.sci.eg; 4 Department of Chemistry, Faculty of Science, Alexandria University, Ibrahimia, Alexandria 21321, Egypt

**Keywords:** Ag/TiO_2_, nanocomposite, antibacterial, photocatalysis, viscose fibers

## Abstract

Multifunctional fibrous surfaces with ultraviolet protection, self-cleaning, or antibacterial activity have been highly attractive. Nanocomposites consisting of silver (AgNPs) and titanium dioxide (TiO_2_ NPs) nanoparticles (Ag/TiO_2_) were developed and coated onto the surface of viscose fibers employing a straightforward pad–dry–cure procedure. The morphologies and elemental compositions were evaluated by scan electron microscopy (SEM), infrared spectra (FTIR), and energy-dispersion X-ray spectra (EDS). The resultant multifunctional textile materials displayed antibacterial and photo-induced catalytic properties. The photocatalyzed self-cleaning properties were investigated employing the photochemical decay of methylthioninium chloride, whereas the antibacterial properties were studied versus *E. coli*. The viscose fibers coated with Ag/TiO_2_ nanocomposite demonstrated improved efficiency compared with viscose fibers coated with pure anatase TiO_2_ nano-scaled particles.

## 1. Introduction

High-performance textiles have significant potential in marketing nano-based functional commodities, such as antibacterial and self-cleaning textiles [1,2,3,4,5]. These nano-based functional textiles can be accomplished by the immobilization of metal and/or metal oxide nanoparticles onto the fabric surface during the finishing process. The great prospects of metal nanoparticles can be effectively employed to provide multifunctional stimulation without deteriorating the exterior properties or negatively affecting the native features of the fibers [6]. Various studies were explored for the usage of Ag^0^, TiO_2_, and ZnO nanoparticles as agents for textile surface modification to provide smart fibers with a variety of distinctive properties, like ultraviolet blocking, self-cleaning, and antibacterial activity [7,8,9]. Different techniques were described recently to tie TiO_2_ nanoparticles into the fiber surface to present self-cleanable products [10]. The photocatalytic performance of TiO_2_ nanoparticles upon irradiation with a UN supply was described. The exposure of TiO_2_ nanoparticles to UV (λ < 388 nm) results in stimulating the electrons of the valence band into the other conduction one to generate holes (h^+^) and electrons (e^−^). Those reactive entities showed a major role in the commencement of a reduction-oxidation course [11]. TiO_2_ nanoparticles have been reported as a high-quality substance in photocatalysis under irradiation with an ultraviolet supply owing to its satisfactory optical properties, chemical/physical stability, non-toxicity, and cheapness [12,13]. Nonetheless, some weakness was linked to the use of TiO_2_ nanoparticles, such as an elevated band-gap (Eg = 3.2 eV). In addition, TiO_2_ nanoparticles can be excited only under irradiation with an ultraviolet supply (λ < 388 nm) to release electrons to conduction band departing holes to the other valence one, limiting their photocatalytic activity under visible or sunlight. Furthermore, the high recombination rate between holes and electrons on TiO_2_ nanoparticles results in less effective photocatalysis [2,3,4,5].

Silver nanoparticles (AgNPs) have been applied as an antimicrobial agent onto a variety of textile substrates in the absence of UV light [14,15]. However, silver nanoparticles can simply influence the colorimetric properties of the treated textile surface by oxidation into the brownish AgO or by aggregation into bigger black microparticles. In addition, silver is a costly metal, and small amounts are ineffective for a variety of realistic products. In order to accomplish the advantageous effects from both Ag^0^ and TiO_2_ nanoparticles and reduce their weaknesses, Ag/TiO_2_ composites were developed by producing AgNPs onto TiO_2_ nanoparticles, employing a variety of methods to enhance the photocatalytic and antimicrobial properties. The deposition of AgNPs can significantly improve the light-induced catalytic activity of TiO_2_ nanoparticles. This could be ascribed to the ability of AgNPs to trap electrons at Schottky bar at each contact area of Ag/TiO_2_ [16,17,18,19]. This results in a decrease in the recombination effect between electrons and holes on the surface of TiO_2_ nanoparticles. Thus, separating the charge was stimulated and the transfer of electrons took place to result in a higher life-time of hole/electron pairs [20,21,22].

Viscose is a significant material for textiles owing to its high resistance to radiation and high stability to body fluids. The improvement of the antimicrobial properties of viscose has been critical for a variety of healthcare purposes. Therefore, various techniques have been reported to improve the antimicrobial properties of viscose fibers [23]. The weak binding of the colloidal nanoparticles to viscose fibers has been a substantial problem that can be overwhelmed by plasma treatment [24]. Plasma curing by etching was employed to activate the fibrous surfaces to induce the creation of polar groups, such as carbonyl, alcohol, carboxyl, and ether, facilitating better binding to nano-scaled particles [5,25]. Herein, we report the synthesis of TiO_2_ NPs and Ag/TiO_2_ nanocomposites as antibacterial and photocatalytic agents and their immobilization onto the surface of viscose fibers via a pad–dry–cure procedure to introduce multifunctional textiles. The morphologies and elemental compositions were evaluated by different analytical techniques. The performance of the Ag/TiO_2_-coated viscose fibers showed an improved efficiency compared with TiO_2_-coated viscose fibers.

## 2. Experimental details

### 2.1. Materials

Viscose fabrics were supplied from Spin and Weaving Misr El-Mahalla Co. (El-Mahalla City, Egypt) Silver(I) nitrate, titanium isopropoxide (TTIP; 97%), acetic acid (65%), silver nitrate (≥99.0%), acetic acid (CH_3_COOH; 96%), nitric acid (HNO_3_; 65%), sodium carbonate (Na_2_CO_3_), and oxalic acid were obtained from Aldrich (Cairo, Egypt). TiO_2_ nanoparticles were synthesized according to the previously reported low temperature sol-gel method [18].

### 2.2. Synthesis of TiO_2_ Nanoparticles

TTIP (2% v-v) was added to a solution of HNO_3_ (1% v-v), CH_3_COOH (10% v-v), and distilled water (DW). The mixture was subjected to stirring for an extra 16 h at 60 °C. After cooling, TiO_2_ NPs were provided by continuously adding Na_2_CO_3_(aq) (5%) until reaching a full sedimentation of TiO_2_ NPs. The generated dispersion was centrifuged (4000 rpm) for 5 min, decanted, washed with DW, and dried at 100 °C over 3 h. The dispersion of the generated TiO_2_ NPs in DW was transparent and stable for several weeks at room temperature. 

### 2.3. Synthesis of Ag/TiO_2_

Ag/TiO_2_ nanocomposite was synthesized utilizing UV technology [26]. Oxalic acid (0.005 mol/L) and AgNO_3_ (0.0002 mol/L) in DW were mixed with a suspension of the above-prepared TiO_2_ NPs (1 g). After stirring for 15 min, the mixture was added to DW (450 mL) with vigorous stirring. The pH was adjusted in the range of 6.8–7.0 using NaOH(aq). The admixture was irradiated with UV supply for 60 min. The admixture was then placed to settle down for 8 h to form a brownish precipitation of Ag^0^/TiO_2_, which was filtered and dried at 120 °C for 3 h to give Ag/TiO_2_.

### 2.4. Deposition of Ag/TiO_2_ onto Plasma-Activated Viscose 

**Figure 1:**
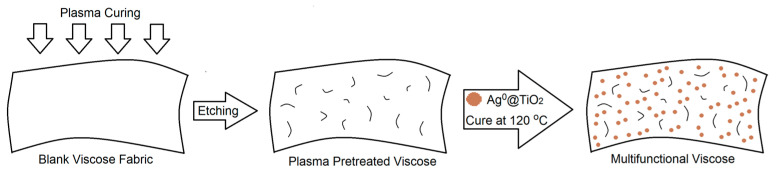
Schematic diagram representing the deposition of Ag^0^/TiO_2_ onto plasma-cured viscose fabric.

As demonstrated in Figure 1, the plasma tool was applied to viscose fibers for 3 min at a power of 400 W and a constant pressure of 3 × 10^−3^ mbar [27]. The above-prepared solutions were then applied to the plasma-activated viscose fibers by the pad–dry–cure process. Both TiO_2_ NPs (0.1 g) and Ag^0^/TiO_2_ (0.1 g) were stirred in DW (150 mL) and homogenized for 45 min under ambient conditions. The plasma-activated fabric (15 cm × 15 cm) was soaked in the prepared solutions for 60 min, and subjected to pad–dry–cure. The viscose was then dried at 90 °C, subjected to curing at 120 °C, and finally rinsed with DW. The binding stability of Ag^0^/TiO_2_ and TiO_2_ onto viscose can be attributed to the electrostatic forces among Ti^4+^ existing on TiO_2_ or Ag^0^/TiO_2_, and the negative charges on the viscose surface. The negative charges on viscose could be attributed to the negatively charged substituents, such as O–O– and –COO– generated by plasma.

### 2.5. Characterization Methods

TEM (JEOL-1230, Akishima, Japan) was applied to inspect the morphology of the prepared TiO_2_ NPs. The morphologies of the coated viscose were explored by Quanta SEM FEG 250 (Brno-Černovice, Czech Republic) linked to EDS (TEAM) to investigate the elemental contents of the viscose coated surface. FT-IR spectra were assessed by Nexus 670 (Nicolet; Watertown, MA, USA). UV/Vis absorption spectra and CIE Lab of the coated viscose were collected by UltraScanPro (Hunter Lab, Reston, VA, USA). The optical band gap was assessed from the absorbance spectrum utilizing Tauc’s equation [ε*hν* = C (*hν*−E_g_)^n^], where E_g_ is the average band gap, ε is molar extinction coefficient, C is a constant, and n relies on the transition type.

### 2.6. Evaluation of Self-Cleaning 

The self-cleaning activity was assessed by the light-induced decay of methylthioninium chloride (MTC) under visible (410 nm) and ultraviolet irradiation (315–380 nm) according to previous literature procedures [28].

### 2.7. Antibacterial Properties 

The antibacterial performance was examined against *E. coli* according to the earlier procedure [29].

### 2.8. Durability Test

To study the durability of the treated viscose against washing, the coated samples (15 cm × 15 cm) were subjected to washing for 10 laundry cycles under AATCC 61:1989 standard procedure. The coated samples were charged in a laundry-o-meter machine, and subjected to washing with a detergent solution (200 mL) at 40 °C for 45 min. Both antibacterial and self-cleaning were assessed as indicators to evaluate the durability of the coated fibers.

## 3. Result and Discussion 

### 3.1. Development of Composite

**Figure 2:**
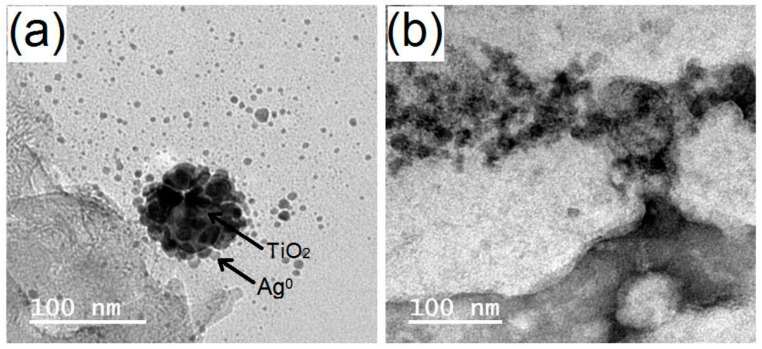
TEM graphs of TiO_2_ (**a**) and Ag^0^/TiO_2_ (**b**).

Ag/TiO_2_ was synthesized under UV technology [19], starting from a mixture of oxalic acid, AgNO_3_, and TiO_2_ NPs (Figure 2). The reaction color was found to change from colorless to a brownish shade under irradiation with ultraviolet light to verify the reduction of silver ions (Ag^+^) to silver metal (Ag^0^) and incorporating AgNPs onto TiO_2_ NPs. The color shift presented a visual verification for the photo-metallization process in the reaction system. Silver ions were initially subjected to cationic adsorption onto the surface of TiO_2_ NPs. When a suspension of TiO_2_ has a pH value <6, the main surface entities become TiOH^2+^, while the main surface entities becomes TiOH^−^ for a suspension of TiO_2_ with a pH value higher than 6. Thus, NaOH(aq) was added for complete deposition of the adsorbed Ag^+^ onto the surface of TiO_2_ NPs to result in the formation of silver(I) oxide (Ag_2_O), which were then reduced to Ag^0^ by an ultraviolet supply. The ultraviolet irradiation has the ability to induce the transfer of free electrons from valence of TiO_2_ NPs to the other conduction band. TiO_2_ NPs comprises negative charges in the presence of Ti-OH^−^, facilitating deposition of Ag^+^ onto its surface. Thus, the photo-induced generated electrons function as reductive agents for Ag^+^ to provide Ag^0^. Production of tiny Ag^0^ crystals could occur by cathode-like reduction or by aggregation of Ag^0^. AgNPs has been known to show an absorbance band attributed to Plasmon effect owing to the interaction of the metallic NPs with UV, leading to oscillation of electrons. The color change of the solution to brown was attributed to the improved absorption at low wavelength owing to surface Plasmon. The reaction mechanism between AgNO_3_ and TiO_2_ is illustrated by the equations described below [30].

Ag^+^ → Ag^+^ (adsorbed onto the surface of TiO_2_ NPs) 2Ag^+^ (adsorbed) + 2OH^−^ → Ag_2_O + H_2_O 2Ag_2_O + *hυ* → 4Ag^0^ + O_2_ TiO_2_ (e-/h^+^) + Ag^+^ → TiO_2_@Ag^0^

**Figure 3:**
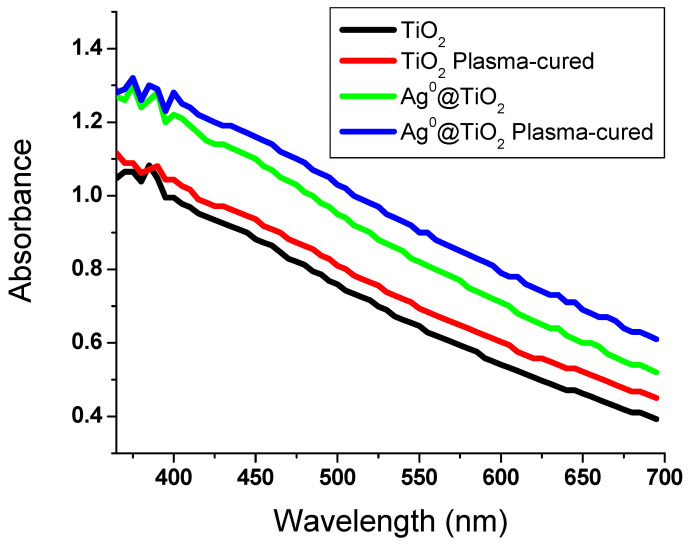
UV/Vis absorption spectral curves of the prepared composites coated onto viscose fibers.

UV/Vis absorption spectra were studied to explore the influence of Ag^0^ on the TiO_2_ optical activity, as illustrated in Figure 3. The absorption spectra of TiO_2_ and Ag^0^/TiO_2_ showed broad absorbance bands with a wavelength maxima <400 nm. This can be attributed to the electron transition in TiO_2_ depending on its energy band gap (~3.12 eV) owing to a charge transfer. The absorbance spectral curves of TiO_2_ were enhanced in Ag^0^/TiO_2_. Obvious variations in absorbance activity of Ag^0^/TiO_2_ were detected in the visible spectrum range as a result of the weak Plasmon effect owing to the low Ag^0^ content on TiO_2_ NPs. This could enhance both surface excitation and electron/hole separation. The absorbance band of Ag^0^/TiO_2_ demonstrated that Ag^0^/TiO_2_ exhibits properties similar to TiO_2_ NPs. The absorbance intensities were observed to exhibit a red shift for Ag^0^/TiO_2_, representing a decrease in TiO_2_ gap. The absorbance spectra showed maximum absorbance wavelengths at 383 and 388 nm for TiO_2_ and Ag^0^/TiO_2_, respectively. This monitored shift in the wavelength and the reduced band gap led to the increase in the light-induced catalytic activity of TiO_2_ NPs in the visible range.

### 3.2. Characterization of Viscose Fibers 

**Figure 4:**
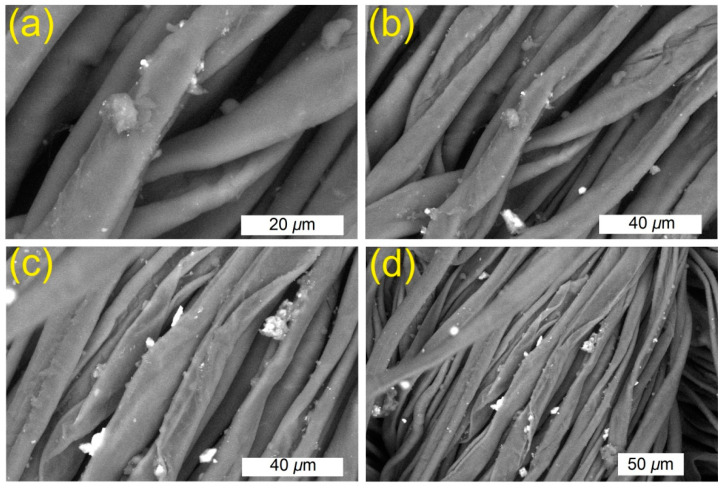
SEM images of TiO_2_ NPs incorporated plasma-activated (**a**,**b**) and Ag^0^/TiO_2_ incorporated plasma-activated (**c**,**d**) fibers.

The morphology of the coated viscose before and after treatment with plasma, as well as plasma-pretreated viscose before and after coating, were studied by SEM as depicted in Figure 4. A surface of moderate smoothness was monitored for plasma-inactivated viscose. Plasma-cured viscose displayed etches on the fiber surface. Irregular nanoparticles were monitored on the surface of the plasma-treated fibers. Decreasing the thickness of the surface layers resulted in improving the rough surface in comparison with pristine fibers. Fibers loaded with TiO_2_ showed irregular and uneven clusters. Fibers coated with Ag^0^/TiO_2_ displayed a skinny film of inconsistent Ag^0^/TiO_2_. No cracking was detected and the small particles were monitored to cover the fibers. The changes in chemical compositions of samples due to plasma-curing and deposition of nanoparticles onto the surface of viscose were explored by EDX. The chemical compositions of blank and plasma-untreated fibers loaded with nanoparticles are summarized in Table 1. Both carbon and oxygen were detected as major contents due to the fabric, whereas Ti and/or Ag were detected as minor contents due to the deposition of TiO_2_ or Ag^0^/TiO_2_ onto the fabric surface. The plasma-cured sample showed a slight increase in the oxygen content due to generating oxygen-containing substituents onto the fiber by oxygen plasma treatment. Plasma curing by etching and oxidation has been employed to activate the fiber surface to induce the creation of substituents [31], such as carbonyl, alcohol, carboxyl, and ether, facilitating strong binding to nano-scaled particles.

**Figure 5:**
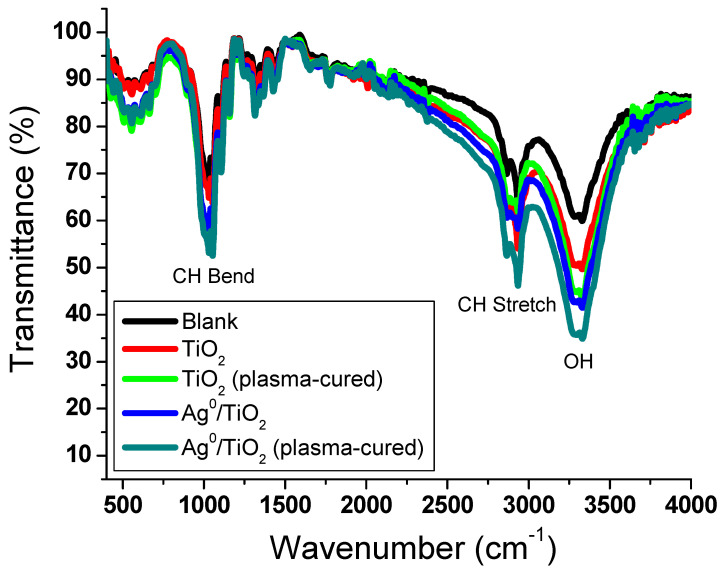
FT-IR spectra of coated viscose fibers.

FT-IR spectra were explored for the coated viscose with and without plasma treatment, as shown in Figure 5. The main characteristic peaks were detected at 3339 cm^−1^ for the hydroxyl group stretch vibration, as well as two peaks at 2932 and 1030 cm^−1^ for the aliphatic C-H stretch and bend vibrations, respectively. No major shifts were detected in the absorbance bands; however, the intensity of the hydroxyl group was found to increase with the increasing deposition of the nanoparticles.

### 3.3. Self-Cleaning Properties 

**Figure 6:**
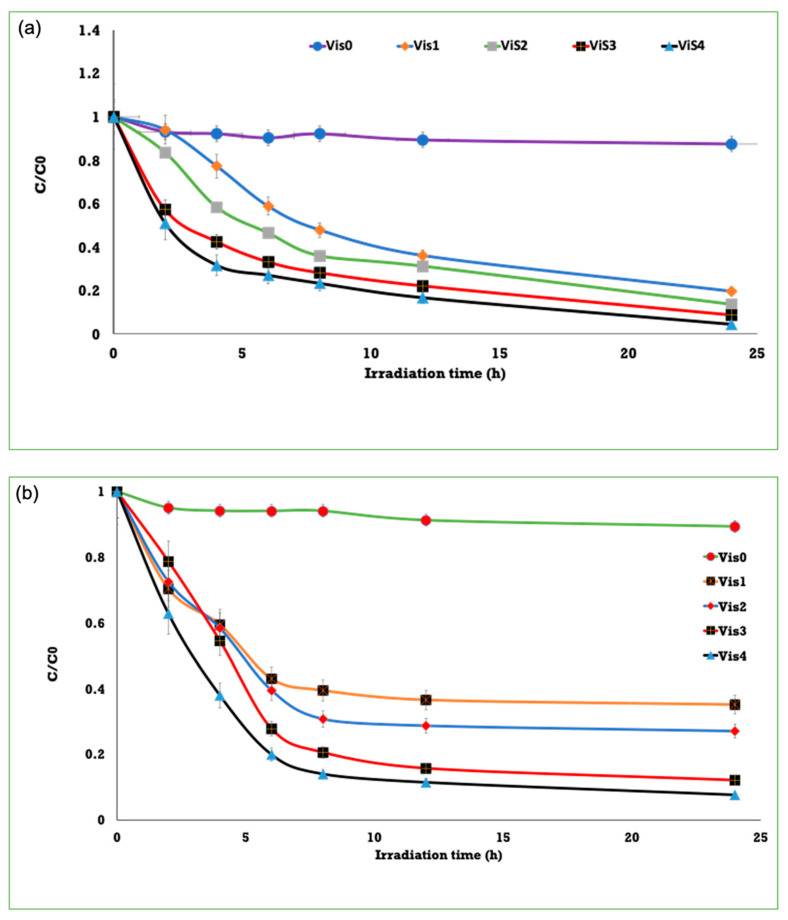
Degradation of MTC on fibers under UV (**a**) and visible (**b**) lights for pristine fibers (Vis_0_), TiO_2_/fibers after wash (Vis_1_), TiO_2_/fibers before wash (Vis_2_), Ag^0^/TiO_2_/fibers after wash (Vis_3_), and Ag^0^/TiO_2_/fibers before wash (Vis_4_).

The reduction potential of MTC is about 0.011 V, whereas the energy level of the conduction band for TiO_2_ is about −0.5 V. Thus, MTC is a suitable model to investigate the photo-induced catalysis process. The self-cleaning performance of TiO_2_ or Ag^0^/TiO_2_ deposited onto viscose fibers could be studied by testing the decomposition of MTC underneath UV and visible lights, as shown in Figure 6. Ultraviolet/visible absorption spectral curves of MTC were collected for the treated viscose under irradiation with UV and visible light over 24 h. The absorbance peak at 665 nm decreased as a result of the degradation of MTC. The self-cleaning activity was tested by exploring the total content (C/C_0_) of MTC as a function of time. The degradation of MTC on the uncoated fibers showed almost no variations under irradiation with either UV or visible daylight to prove that the uncoated fibers do not exhibit any light-induced decay ability. The deposition of TiO_2_ NPs onto plasma-activated fibers proved an improvement in photo-induced degradation of MTC under UV light. However, this photo-induced degradation of MTC was incomplete. The deposition of TiO_2_ onto plasma-pretreated fibers displayed a negligible photo-induced degradation under visible light owing to the adsorption and diffusion of MTC within the coated viscose. The integration of the nanocomposite into the plasma-pretreated fibers induced a total photoinduced degradation of MTC under ultraviolet and visible light, as the blue shade was monitored neither on the coated fibers nor in solution to prove a complete photo-induced degradation of MTC. The photo-induced degradation of MTC for fibers coated with Ag^0^/TiO_2_ demonstrated improved activity in comparison with TiO_2_ NPs, proposing that the inclusion of AgNPs onto the surface of TiO_2_ is an efficient approach. The photo-induced degradation rate for TiO_2_ and Ag^0^/TiO_2_ coated onto viscose fibers decreased with washing. Nonetheless, they persisted higher than the plasma-inactivated viscose fibers. This proposed higher adhesion of particles onto plasma-activated viscose. After washing, the light-induced decay of methylthioninium chloride (MTC) for Ag^0^/TiO_2_ deposited onto viscose fibers was lower than the case of the viscose fibers coated with TiO_2_ under visible/UV light. Thus, the nanocomposite enhanced the self-cleaning activity of viscose as a beneficial effect of silver on the light-induced catalysis of TiO_2_.

### 3.4. Antibacterial Activity 

**Figure 7:**
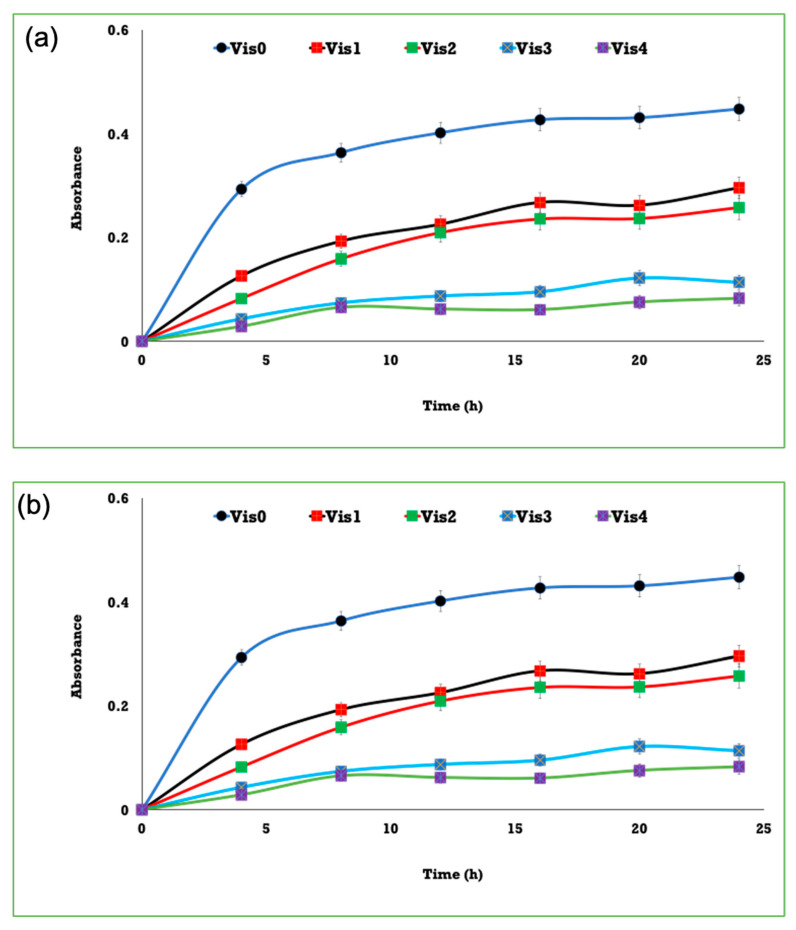
Activity of coated viscose fibers against *E. coli*; Vis_0_ is pristine viscose, Vis_1_ is 10^3^ (**a**) or 10^6^ (**b**) bacterial density, Vis_2_ is NPs/fibers, Vis_3_ is nanocomposite/fibers following washing, and Vis_4_ is nanocomposite/fibers prior to washing.

The antibacterial properties of plasma-cured and coated viscose were examined against *E. coli* by measuring optical density (OD) at 620 nm versus time, as shown in Figure 7. OD was found to improve, reflecting the decrease in the quantity of growing bacteria in the tested sample. Both blank and TiO_2_ coated viscose fibers displayed no inhibition. The viscose fibers coated with Ag^0^/TiO_2_ showed antibacterial properties at all contents, yet followed by washing to confirm the positive effect of loading Ag^0^ onto TiO_2_. AgNPs have been described to exhibit a broad of activity against a variety of pathogens. It has been recognized that the increase in surface area results in improved antibacterial properties [32].

## 4. Conclusions

Multifunctional viscose fibers coated with Ag/TiO_2_ nanocomposite were developed by the simple pad–dry–cure technology. The synthesis, characterization, and use of nanocomposite as an antibacterial and light-induced self-cleaning agent were explored. Ag/TiO_2_ was prepared using a double-stage procedure of sol–gel TiO_2_ synthesis, followed by depositing of Ag^0^ onto the surface of TiO_2_ by ultraviolet irradiation. The deposition of Ag^0^/TiO_2_ onto plasma-pretreated viscose fibers was accomplished using the facile pad–dry–cure technology. Ag^0^/TiO_2_ displayed better absorption in the visible spectrum and higher antibacterial activity and light-induced catalysis in comparison with plasma-activated viscose coated with TiO_2_. This considerable improvement in antibacterial and self-clean properties could be attributed to AgNPs deposited onto the surface of TiO_2_. The current study presented a good strategy to produce Ag/TiO_2_ composite with the ability to impart antibacterial, self-cleaning photo-induced catalytic properties to plasma-cured fibers, under irradiation with UV/visible lights to make this Ag^0^/TiO_2_ nanocomposite potentially practical as a multifunctional agent for a variety of applications, such as medical clothing. 

## Figures and Tables

**Table 1:** Elemental contents of viscose fibers.

Sample	C	O	Ti	Ag
Blank	62.12 ± 1.3	37.88 ± 1.2	0	0
Plasma-activated	61.71 ± 1.1	38.29 ± 1.0	0	0
Plasma-inactivated (TiO_2_)	59.44 ± 1.6	38.91 ± 1.1	1.65 ± 0.1	0
Plasma-activated (TiO_2_)	57.14 ± 1.4	39.12 ± 1.6	3.74 ± 0.3	0
Plasma-inactivated (Ag^0^/TiO_2_)	59.03 ± 1.0	38.73 ± 1.3	1.72 ± 0.1	0.52 ± 0.1
Plasma-activated (Ag^0^/TiO_2_)	56.11 ± 1.2	39.43 ± 1.2	3.34 ± 0.2	1.12 ± 0.1

## Data Availability

The data presented in this study are available on request from the corresponding author.
